# Challenges to Contemporary Wide Complex Tachycardia Criteria: A Single-center Case Series of 1:1 Atrial Flutter

**DOI:** 10.19102/icrm.2024.15102

**Published:** 2024-10-15

**Authors:** Garrett M. Snipes, Jacob N. Blackwell, Prashant D. Bhave

**Affiliations:** 1 Department of Internal Medicine, Section of Cardiovascular Medicine, Wake Forest University School of Medicine, Winston-Salem, NC, USA

**Keywords:** Aberrant conduction, atrial flutter, tachyarrhythmia, wide complex tachycardia

## Abstract

Atrial flutter with 1:1 atrioventricular conduction is a rare cause of wide complex tachycardia, which presents a diagnostic challenge. This report describes a series of eight cases of 1:1 atrial flutter compiled during 2018–2022. The cases in this report include patients without class 1 anti-arrhythmic use or pre-excitation.

## Introduction

Timely differentiation of the underlying mechanism of wide complex tachyarrhythmias remains a challenge. Atrial flutter with 1:1 atrioventricular (AV) conduction is a rare cause of wide complex tachycardia (WCT) first described in 1915 by Dr. Thomas Lewis.^1^ At ventricular rates frequently exceeding 240 bpm, 1:1 atrial flutter often displays aberrant conduction.^2^ The prevalence of 1:1 AV conduction has been cited at 8% among patients with atrial flutter.^3^ Prior publications describe 1:1 atrial flutter in the context of class 1c anti-arrhythmic therapy or underlying pre-excitation.^3–5^ Younger age, a short P–R interval while in sinus rhythm, the absence of an underlying heart disease, and a history of atrial fibrillation have also been described as risk factors for 1:1 AV conduction of atrial flutter.^3^ This report includes a series of eight cases of 1:1 atrial flutter in patients without class 1 anti-arrhythmic use or pre-excitation.

Cases were identified among patients seen during 2018–2022 at our institution. **Table 1** displays a summary of case details and the assessment of electrocardiogram (ECG) tracings according to the Brugada, Vereckei, and Kindwall left bundle branch block (LBBB) criteria. The collection of informed consent was not required for this study.

## Cases

### Case 1

**Figure 1:**
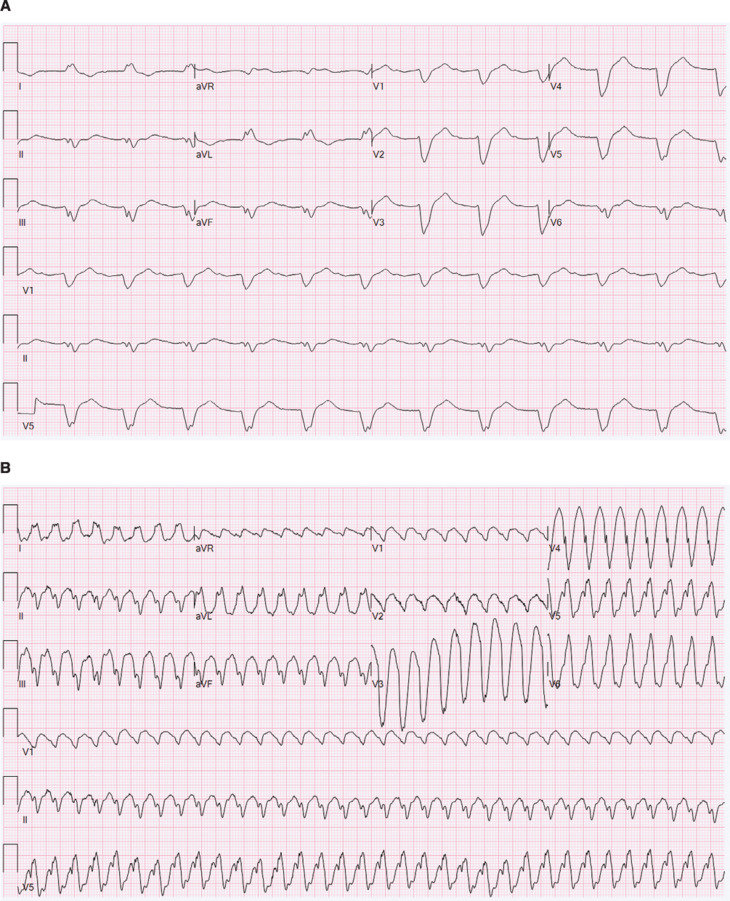
Case 1 electrocardiograms. **A:** Baseline conduction disease with left bundle branch block and first-degree atrioventricular block prior to clinical presentation. **B:** Atrial flutter with 1:1 aberrant conduction at the time of presentation.

A 62-year-old man with a history of LBBB, cocaine abuse, alcohol abuse, heart failure with reduced ejection fraction, paroxysmal typical atrial flutter, and atrial fibrillation presented to the hospital with palpitations. His baseline ECG is shown in **Figure 1A**. The ECG demonstrated a WCT with a rate of 210 bpm **(Figure 1B)**. His lab work was notable for urine drug screen positive for cocaine metabolites. Intravenous (IV) amiodarone was initiated in the emergency department, and the patient underwent synchronized cardioversion. He has not had any clinically apparent recurrence of atrial flutter as of the 6-month follow-up.

### Case 2

A 29-year-old man with a history of LBBB, congenital aortic stenosis treated with a remote Ross procedure, and subsequent mechanical aortic valve and bioprosthetic pulmonic valve replacement presented to the emergency department with palpitations. His ECG demonstrated a WCT with a rate of approximately 280 bpm. He was promptly sedated and cardioverted to sinus rhythm. The patient underwent electrophysiology (EP) testing, which revealed inducible typical atrial flutter at a rate matching that of the presenting WCT. He underwent a cavotricuspid isthmus (CTI) ablation for typical atrial flutter and remained without recurrence at 1 year of follow-up.

### Case 3

**Figure 2:**
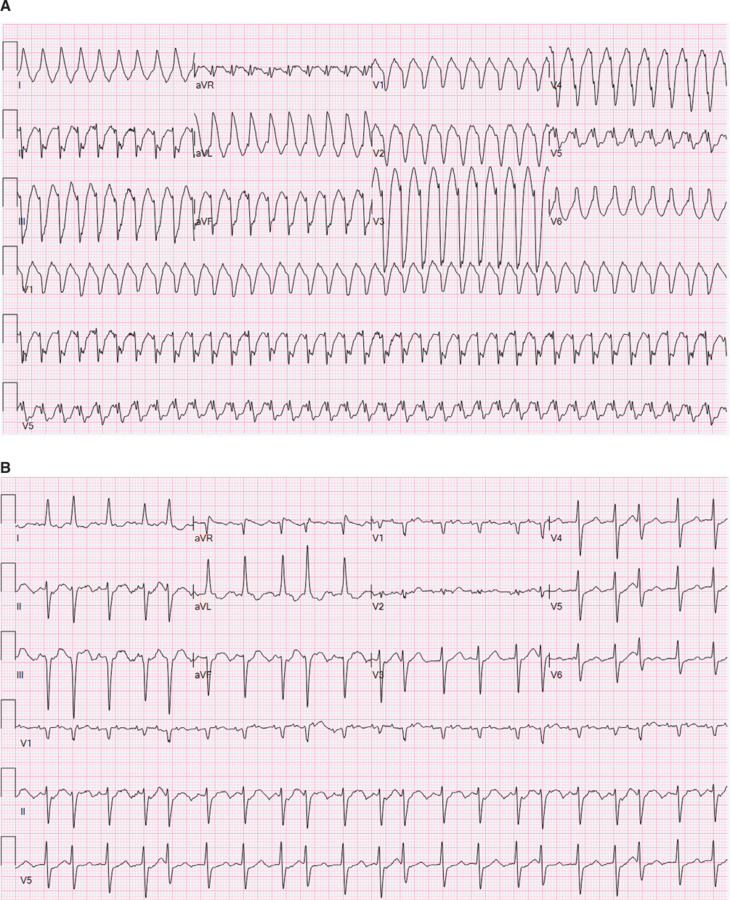
Case 3 electrocardiograms. **A:** Typical atrial flutter with 1:1 atrioventricular conduction with a ventricular response rate of 224 bpm and a QRS duration of 188 ms. **B:** Typical atrial flutter with variable atrioventricular block with the initiation of intravenous amiodarone. Narrowing of the QRS complex was noted with slowing of the ventricular response rate.

A 59-year-old man with no pertinent history presented with dyspnea and fatigue. His ECG demonstrated a WCT with a rate of 220 bpm **(Figure 2A)**. He was treated with an amiodarone infusion. With subsequent slowing of the ventricular rate, the ECG demonstrated typical atrial flutter with variable AV conduction **(Figure 2B)**. He underwent a CTI ablation. He remained without recurrence at the 3-year follow-up.

### Case 4

A 70-year-old man with paroxysmal atrial fibrillation and atrial flutter with prior pulmonary vein isolation and CTI ablation presented to the emergency department with palpitations and dyspnea. His ECG demonstrated a regular WCT with a rate of 226 bpm. His ventricular rate spontaneously slowed to 110 bpm, which revealed an atypical atrial flutter. He underwent transesophageal echocardiography cardioversion and was started on dofetilide and an adjusted dose of metoprolol. The patient elected for pharmacologic rhythm control. At the 1-year follow-up, he remained without clinically apparent recurrence of his atypical atrial flutter.

### Case 5

A 45-year-old man with no past medical history presented with palpitations, chest pressure, and dyspnea at rest. His ECG demonstrated a WCT with a rate of 240 bpm. Synchronized cardioversion was performed, which resulted in conversion to atrial fibrillation. A subsequent EP study resulted in no inducible ventricular tachycardia (VT). Non-sustained atypical atrial flutter was induced. Isoproterenol infusion with rapid atrial pacing produced a pattern of aberrancy, which matched the QRS morphology of his presenting WCT. He was discharged on 100 mg of atenolol daily for AV nodal blockade. Subsequently, this patient was lost to follow-up.

### Case 6

A 62-year-old man with a history of typical atrial flutter with prior CTI ablation, atypical flutter, and tachycardia-induced cardiomyopathy with recovered ejection fraction presented with chest pressure, palpitations, and dyspnea at rest. His ECG demonstrated a WCT with a rate of 252 bpm. The patient underwent synchronized cardioversion with subsequent conversion to atrial fibrillation. During the EP study, the patient’s clinical arrhythmia was successfully induced with isoproterenol infusion and atrial burst pacing, which was consistent with counterclockwise typical atrial flutter. A gap was identified in the proximal CTI from the mid-portion to the inferior vena cava. A successful ablation of the gap with confirmation of bidirectional block was performed. No VT or ventricular fibrillation could be induced. Given the patient’s history of tachycardia-induced cardiomyopathy, atrial fibrillation, and the recurrent nature of the atrial flutter, the patient was discharged on 200 mg of amiodarone. At the 1-year follow-up, he remained without recurrent atrial flutter.

### Case 7

A 30-year-old man with trisomy 21 complicated by a transitional AV canal defect with remote atrial septal defect repair and aortic valve repair with the Ross procedure presented with syncope and palpitations. His ECG demonstrated a WCT with a rate of 290 bpm. On review of telemetry, the waveform varied from a wide to narrow complex associated with the ventricular rate and demonstrated variable AV conduction suggestive of atrial flutter. Due to presyncope symptoms at higher ventricular rates, the patient was electrically cardioverted. He was subsequently referred for a CTI ablation in the outpatient setting 2 weeks after the initial presentation. At the 3-month follow-up after ablation, he remained free of clinically apparent arrhythmias.

### Case 8

A 61-year-old man with paroxysmal atrial fibrillation not on anticoagulation presented with a sudden onset of palpitation and fatigue. His ECG demonstrated a WCT with a rate of approximately 200 bpm. IV diltiazem, serial doses of adenosine, and cardioversion were attempted in the field by emergency medical services without the restoration of sinus rhythm. On arrival, his ECG demonstrated a WCT with a rate of 255 bpm. The QRS morphology was similar to the patient’s known right bundle branch block noted on prior ECGs, but the QRS had widened to 170 ms. The patient was treated with a diltiazem infusion, which resulted in the slowing of AV conduction of the atrial flutter and QRS narrowing. The patient underwent a successful CTI ablation but developed atrial fibrillation, which he was cardioverted out of at the end of the case. The patient was ultimately placed on oral anticoagulation and metoprolol at discharge for the management of paroxysmal atrial fibrillation. He maintained sinus rhythm at the 1-month follow-up.

## Discussion

This case series of eight patients demonstrates the heterogeneous clinical presentation and electrocardiographic appearance of atrial flutter with 1:1 AV conduction. CTI-dependent and atypical flutter were both documented.

Class 1 anti-arrhythmics slow intra-atrial conduction, which can promote 1:1 AV conduction and paradoxically increase ventricular activation in the setting of atrial flutter.^5,6^ Prior to this series, there are only limited data describing 1:1 AV conduction of atrial flutter without the influence of anti-arrhythmic therapy on the flutter cycle length.^7,8^ Pre-excitation has also previously been reported as a predisposing factor for 1:1 AV conduction in atrial flutter.^9^ The cases described herein all occurred in patients without an accessory pathway, demonstrating that native AV nodal function has the capacity to maintain 1:1 AV conduction during atrial flutter.

The early clinical courses and ECG tracings of the cases described in this report highlight how atrial flutter with 1:1 AV conduction can be mistaken for VT. Sample ECG tracings from the cases are seen in **Figures 1A and 1B**. Well-established WCT differentiation tools were used to examine the tracings from each case. The results are displayed in **Table 1**. In the majority of cases, both the Brugada and Vereckei criteria incorrectly identified the underlying rhythms as VT. The Kindwall LBBB criteria for VT were not applicable to the majority of cases due to their QRS morphology. Of the cases where the criteria could be applied, two arrhythmias were correctly identified as supraventricular in origin. The patients in cases 2 and 7 both had a history of congenital heart disease. It is important to note the established limitations of both the Brugada and Vereckei criteria in this patient population.^10^ The overall poor performance of these WCT assessment tools underlines how atrial flutter with 1:1 AV conduction and aberrant ventricular conduction can be easily misidentified as VT. This highlights a key challenge for physicians in the management of these arrhythmias.

Given the shortcomings of traditional ECG rhythm–assessment algorithms, often, an EP study is necessary to clarify the source of the patient’s tachyarrhythmia once they are stabilized. The cases in this study identified as having a typical CTI-dependent atrial flutter and treated with catheter ablation demonstrated high freedom from arrhythmia at follow-up.

## Conclusion

Atrial flutter with 1:1 AV conduction is a clinical entity that can occur across a wide array of clinical scenarios, even in the absence of pre-excitation or class 1 anti-arrhythmics. In the presence of aberrant ventricular conduction, atrial flutter with 1:1 AV conduction can easily be misdiagnosed as VT. Atrial flutter with 1:1 AV conduction as a cause of WCT may be under-recognized given its heterogeneous clinical and electrophysiologic characteristics.

## Figures and Tables

**Table 1:** Summary of Case Characteristics and Rhythm Adjudication with Vereckei, Brugada, and Kindwall Algorithms

Case	Sex	Age (Years)	Presentation	Flutter Location	Baseline Conduction Abnormality	Management	Vereckei	Brugada	Kindwall (LBBB Only)
**1**	M	62	Palpitations	Multiple circuits	LBBB First-degree AV block	Amiodarone and cocaine abstinence	VT	VT	VT
**2**	M	29	Palpitations	CTI-dependent	LBBB	CTI ablation	SVT	VT	SVT
**3**	M	59	Dyspnea and fatigue	Atypical	LAFB	CTI ablation	VT	VT	N/a
**4**	M	70	Dyspnea and palpitations	Atypical	First-degree AV block	Cardioversion, metoprolol, dofetilide	VT	VT	N/a
**5**	M	45	Palpitations, chest pressure, and dyspnea	Atypical	None	Atenolol	VT	VT	N/a
**6**	M	62	Palpitations, chest pressure, and dyspnea	CTI-dependent	RBBB LAFB	Repeat CTI ablation and amiodarone	VT	SVT	N/a
**7**	M	30	Palpitations and syncope	CTI-dependent	None	CTI ablation and metoprolol	VT	VT	N/a
**8**	M	61	Palpitations and fatigue	CTI-dependent	RBBB	CTI ablation	SVT	SVT	N/a

*Abbreviations:* AV, atrioventricular; CTI, cavotricuspid isthmus; LAFB, left anterior fascicular block; LBBB, left bundle branch block; n/a, electrocardiogram tracings where the Kindwall LBBB criteria were not applicable; RBBB, right bundle branch block; SVT, supraventricular tachycardia; VT, ventricular tachycardia.
